# Improving the diagnostic accuracy of acute myocardial infarction with the use of high-sensitive cardiac troponin T in different chronic kidney disease stages

**DOI:** 10.1038/srep41350

**Published:** 2017-02-01

**Authors:** Hongliu Yang, Jing Liu, Han Luo, Xiaoxi Zeng, Xi Tang, Liang Ma, Hongxia Mai, Shenju Gou, Fang Liu, Ping Fu

**Affiliations:** 1 Division of Nephrology, Kidney Research Laboratory, West China Biostatistics and Cost Benefit Analysis Center of West China Hospital, West China Medical School, Sichuan University, Chengdu, China; 2 Division of Thyroid Surgery, West China Hospital, West China Medical School, Sichuan University, Chengdu, China

## Abstract

High-sensitive cardiac troponin T (hs-TnT) is a critical biomarker in diagnosis of acute myocardial infarction (AMI). However, CKD individuals usually have elevated hs-TnT even in the absence of AMI. Our study aimed to explore the optimal cutoff-value of hs-TnT and further to improve diagnostic accuracy of AMI in CKD patients. Clinical data of 489 patients were collected from the maintained database between September 2010 and June 2014. CKD patients with AMI were assigned to CKD+AMI group and CKD patients without AMI were assigned to CKD group. Receiver operating characteristic curves were utilized to derive the optimal cutoff-value. In CKD+STEMI and CKD group, hs-TnT was increased with descending eGFR. In CKD+NSTEMI group, hs-TnT showed an upward trend with increasing SYNTAX Score. In patients with CKD+STEMI, hs-TnT was significantly correlated with SYNTAX Score in CKD stage 2, stage 4 and in total. In CKD patients, the optimal cutoff-value of hs-TnT for diagnosis of AMI was 129.45 ng/l with 75.2% sensitivity and 83.2% specificity. The cutoff-value appeared to be hs-TnT level of 99.55ng/l in CKD stage 3, 129.45 ng/l in CKD stage 4, 105.50 ng/l in CKD stage 5 and 149.35 ng/l in dialysis patients, respectively. In different stages of CKD, eGFR-range-specific optimal cutoff-values should be considered.

Patients with CKD are at high risk of cardiovascular disease (CVD), have worse prognosis with higher mortality after AMI, and have higher risk of recurrent AMI, heart failure and sudden cardiac death^1^. Early diagnosis and intervention have been considered the cornerstone of improving prognosis of AMI in these individuals. Cardiac troponinT (cTnT), a critical biomarker in the diagnosis of AMI, is a low-molecular-weight protein that forms part of the troponin complex acting as an integral component in the myofibrillar contractile apparatus. Loss of integrity of cardiac myocyte membranes causes release of cardiac troponins into circulation, which can be detected by highly sensitive assays developed for cTnT to diagnose AMI^2^. However, elevation of serum cTnT concentration can occur in the absence of AMI, especially in CKD. Numerous data have proven that elevated hs-TnT levels are common in CKD patients^3^,^4^,^5^ and in end stage renal disease (ESRD) patients this tendency has been observed in 20–90% of subjects^6^. With the progression of CKD, subjects with renal dysfunction have gradually elevated troponin levels^7^.

KDIGO guidelines^1^ recommend that in people with GFR <60 ml/min/1.73 m^2^, serum troponin should be interpreted with caution in the diagnosis of acute coronary syndrome. Nevertheless, there is limited data on “expected” value of cTnT for diagnosis of AMI in CKD individuals. Current reference values of cardiac biomarkers to diagnose AMI were based on healthy individuals and were proven to have poor diagnostic accuracy among CKD patients^8^,^9^, which has brought great challenges for clinics. Recently, Twerenbold and colleagues reported optimal cutoff level of more sensitive cardiac troponin assay for diagnosis of AMI in patients with renal dysfunction. However, in this first and only study that explored the “expected” cutoff-value, “patients with renal dysfunction” were assumed as a whole and no subgroup of different stages of renal function was analyzed. This serious clinical problem desperately requires studies to explore the hs-TnT cutoff-value focusing on different CKD stages. Therefore, our study attempted to find the optimal cutoff-value of hs-TnT for the diagnosis of AMI in different stages of CKD, and thus to improve diagnostic accuracy.

## Method and Materials

### Study population

Clinical characteristics and laboratory tests from the inpatient database of West China Hospital through September 2010 to June 2014 were collected. CKD patients who had a complaint description of chest pain and tested serum hs-TnT with an onset or peak within the last 12 hours were identified and assigned to two groups: CKD+AMI group and CKD (non-AMI) group. Diagnoses of CKD were reconfirmed by two independent nephrologists according to 2012KDIGO guidelines^1^ and all AMIs were adjudicated by two independent cardiologists reviewing all available medical records: patient history, physical examination, results of laboratory testing (including hs-TnT levels), radiographs, ECG, echocardiographs and coronary angiographs according to 2012 “Third universal definition of myocardial infarction”^10^. 302 patients meeting the criteria of CKD and AMI simultaneously were recruited and identified as CKD+AMI group. 187 patients without AMI and but with the diagnostic criteria of CKD were identified as CKD group. Patients were excluded for previous myocardial infarction and with diseases that may cause hs-TnT elevation other than AMI^11^ such as hypertensive crisis, tachy- or bradyarrhythmias, pulmonary embolism, severe pulmonary hypertension, myocarditis, acute neurological disease (stroke, or subarachnoid haemorrhage), aortic dissection, aortic valve disease or hypertrophic cardiomyopathy, cardiac contusion, ablation, pacing, cardioversion, endomyocardial biopsy, hypothyroidism, apical ballooning syndrome (Tako-Tsubo cardiomyopathy), infiltrative diseases (amyloidosis, haemochromatosis, sarcoidosis, sclerodermia), drug toxicity (e.g. adriamycin, 5-fluorouracil, herceptin, snake venoms), burns, rhabdomyolysis and Multiple Organ Dysfunction Syndrome. All experimental protocols were approved by West China Hospital. The methods were carried out in accordance with the approved guidelines. Informed consent was obtained from all subjects.

### Clinical and laboratory examination

All the data was extracted from medical recordings. Complete physical examination was collected, and comorbidities like diabetes mellitus (DM), hypertension and lifestyle factor for example cigarette smoking were recorded. DM was diagnosed in our center in accordance with OGTT-based WHO criteria^12^ and hypertension was defined as a systolic blood pressure of 140 mmHg or more, or a diastolic blood pressure of 90 mmHg or more, or taking antihypertensive medication^13^. Weight and height were measured in all subjects, and body mass index (BMI) was calculated as weight (kg)/height^2^ (m^2^). Estimated glomerular filtration rate (eGFR) was calculated from CKD-EPI creatinine equation in accordance with 2012 KDIGO guidelines^1^. hs-TnT was measured using a fifth-generation electrochemiluminescence assay (Elecys, Cobas e602 analyzer, Roche Diagnostics) and according to the manufacturer of the assay, the stated limit of detection is 5 ng/l, the analytical range is 3–10000 ng/l, and the upper reference limit (URL) (99^th^ percentile) in the normal population is 14 ng/l. Brain natriuretic peptide (BNP) was measured with Elecsys Roche Diagnostic commercial kits on a semiautomatic analyzer (Elecsys-2010, Roche Diagnostics, Germany). The severity of infarction was assessed using a SYNTAX Score based on coronary angiogram. All angiographic variables pertinent to SYNTAX Score calculation were computed by two independent experienced cardiologists who were blinded to the current study on angiograms. Hemoglobin(Hb), total leucocyte(WBC), platelet(PLT), blood urea nitrogen (BUN), cystatin-C, creatinine(Cr) and urine test including protein, red blood cell, white blood cell of urea were measured by automated biochemical analyzer. For patients on dialysis, only blood tests during the dialysis interval were collected.

### Statistical methods

Measurement data were analyzed with Normality test. Data that fit the normal distribution were expressed as the mean ± standard deviation and analyzed by t test. Data with a skewed distribution were reported as median and interquartile range (IQR) and analyzed by Mann–Whitney test. Enumeration data were expressed as percentage and analyzed with chi-square test. The correlation between variables and hs-TnT was assessed by Spearman rank correlation test. Effect of those variables on hs-TnT were evaluated by a multiple linear regression model. The cutoff-value of hs-TnT to diagnose AMI was calculated by Receiver operating characteristic (ROC) curve. Diagnostic accuracy was quantified by the area under the curve (AUC). All statistical analysis was performed with SPSS 19.0 (Chicago, IL, USA). P-values < 0.05 were considered statistically significant.

## Results

489 patients (302 in CKD+AMI group and 187 in CKD group) were enrolled. The baseline characteristics of the participants in each group are shown in Table 1. Mean age of the patients was 70.68 years and 280(57.26%) of the participants were male. Subjects in CKD+AMI group were older and had higher proportion of cigarette smokers and higher Hb concentration. 117 patients had coronary angiography (111 in CKD+AMI group and 6 in CKD group). In CKD+AMI group, 181 patients were diagnosed of ST-elevation myocardial infarction (STEMI) whereas 121 of non-ST-elevation myocardial infarction (NSTEMI).

Baseline hs-TnT concentrations in CKD+AMI and CKD group were 668.20(129.40–2716.25) ng/l and 52.60(28.45–97.75) ng/l, respectively. Patients of CKD+AMI group had higher hs-TnT level compared with CKD group (P < 0.001). hs-TnT concentrations and SYNTAX scores of different AMI type under different eGFR categories are shown in Table 2. In each eGFR group, hs-TnT in CKD+AMI group was higher than in CKD group. And hs-TnT was higher in CKD+STEMI group than in CKD+NSTEMI group. In CKD+STEMI and CKD (non-AMI) group, hs-TnT concentration was increased with descending eGFR. While in CKD+NSTEMI group, hs-TnT showed an upward trend with increasing SYNTAX Score.

The relationship between hs-TnT and SYNTAX Score of CKD patients with different types of AMI under each eGFR category is shown in Table 3. In patients with CKD+STEMI, there was significant correlation between hs-TnT and SYNTAX Score in CKD stage 2(60 ≤ eGFR ≤ 89 ml/min/1.73 m^2^), stage 4(15 ≤ eGFR ≤ 29 ml/min/1.73 m^2^) and in total. However, in patients with CKD+NSTEMI, there was no significant correlation between hs-TnT and SYNTAX Score in all of the eGFR categories.

In patients with CKD+AMI, hs-TnT concentration was significantly higher in smoking patients [1130.00(161.15–3315.25)] compared with non-smoking patients [454.80(107.28–2322.50)] (P = 0.011). In addition, hs-TnT concentration was significantly higher in STEMI patients [1133.00(147.75–4013.00)] compared with NSETMI patients [283.00(105.55–1211.00)] (P < 0.001) (Table 4). The above results indicated smoking and ST segment elevation may be key factors influencing hs-TnT concentration. In Spearman rank correlation test, hs-TnT had a positive correlation with BNP (r = 0.403, P < 0.001) and Cr (r = 0.134, P = 0.020) in CKD+AMI patients. Multiple linear regression analysis showed that ST segment elevation (β = 0.305, P < 0.001)and BNP(β = 0.364, P < 0.001) had unique associations with hs-TnT concentration (Table 5).

**Figure 1:**
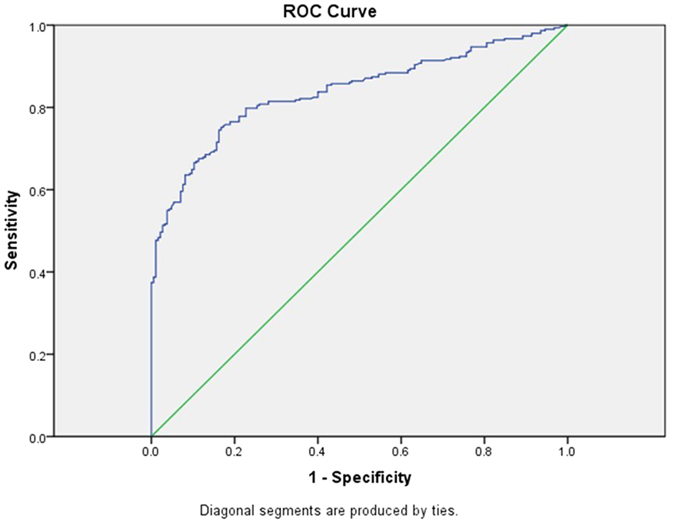
Receiver-operating characteristic (ROC) curve for diagnosis of AMI in the total study population.

**Figure 2:**
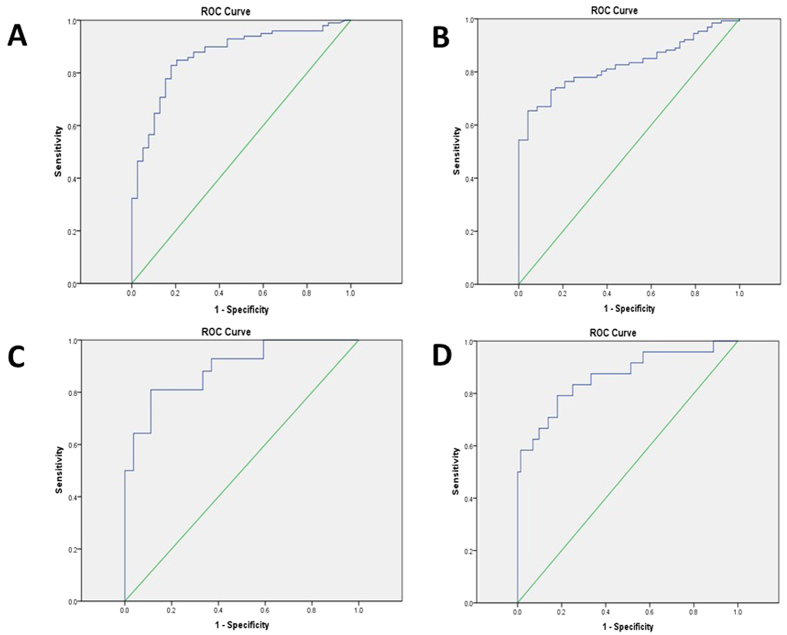
(**A**) Receiver-operating characteristic (ROC) curve for diagnosis of AMI in CKD stage 3 patients. (**B**) ROC curve for diagnosis of AMI in CKD stage 4 patients. (**C**) ROC curve for diagnosis of AMI in CKD stage 5 non-dialysis patients. (**D**) ROC curve for diagnosis of AMI in dialysis patients.

Via ROC curve, optimal cutoff-value for diagnosis of AMI in CKD patients appeared to be an hs-TnT level of 129.45 ng/l with 75.2% sensitivity and 83.2% specificity. AUC was 0.839 (95%CI 0.804–0.873) (Fig. 1). Optimal cutoff-values of hs-TnT varied in different eGFR categories (Table 6 and Fig. 2). In CKD stage 3 patients, the optimal cutoff-value for diagnosis of AMI was 99.55 ng/l with 82.8% sensitivity and 82.1%specificity. AUC was 0.868 (95%CI 0.803–0.932) (Fig. 2A). In CKD stage 4 patients, the optimal cutoff-value for diagnosis of AMI was 129.45 ng/l with 73.2% sensitivity and 85.4%specificity. AUC was 0.827(95% CI 0.768–0.886) (Fig. 2B). In CKD stage 5 non-dialysis patients, the optimal cutoff-value for diagnosis of AMI was 105.50 ng/l with 81.0% sensitivity and 88.9%specificity. AUC was 0.892(95% CI 0.819–0.965) (Fig. 2C). In dialysis patients, the optimal cutoff-value of AMI was 149.35 ng/l with 79.2% sensitivity and 81.9%specificity. AUC was 0.865(95% CI 0.769–0.960) (Fig. 2D).

## Discussion

In the present study, we compared hs-TnT levels in CKD and CKD+AMI patients, as well as in the subgroups of different eGFR categories and AMI types. Further, we derived cutoff-values from ROC curves to indicate optimal hs-TnT level for diagnose of AMI in CKD individuals.

Although the 99^th^ percentile of healthy individuals is the undisputed reference value to diagnose AMI according to the latest universal definition of AMI^10^, optimal clinical decision levels or cutoff levels at presentation to CKD patients may well differ from the 99^th^ percentile of healthy individuals as they have elevated baseline hs-TnT level even in the absence of AMI. A previous study had tested this (99^th^ percentile) cutoff value in patients with renal insufficiency. The diagnostic accuracy was poor with sensitivity of 74%, specificity of 31% and AUC of only 0.535^8^. Twerenbold’s recent study^14^ suggested a cutoff-value of hs-TnT level of 29.5 ng/l for AMI in kidney disease patients, however, 88% of their AMI patients were CKD stage 3. Due to different cutoff-values derived from ROC curves in different eGFR categories in our study, it is notable that hs-TnT level for diagnosis of AMI should be considered according to renal function.

In our study, the cutoff-values were not increased monotonically with decreasing of eGFR. One possible explanation may be with the decreasing of eGFR, CKD patients were more insensitive to chest pain^15^, resulting in missed diagnosis of asymptomatic AMI patients with CKD. In addition, different proportion of AMI patients in each eGFR category may also contribute to these results. The cutoff-value in CKD stage 4 group was the closest to the total study population. This may be due to the larger proportion (40%) of CKD stage 4 patients included in the study population. Besides, dialysis may separately influence hs-TnT concentrations^16^. Therefore, blood tests of dialysis patients during dialysis interval were collected in our study. Furthermore, subgroup analysis of dialysis patients were performed to eliminate this influence.

Our data of an increased hs-TnT level in subjects with lower eGFR CKD non-AMI patients is in agreement with the literature^17^. To our knowledge, the association of hs-TnT with eGFR and severity of infarction in CKD+AMI patients has not been studied specifically. Our study highlighted that hs-TnT level may correlate with multiple factors and their corresponding interaction, especially eGFR, type of AMI and SYNTAX Score. In CKD+STEMI and CKD+NSTEMI patients, hs-TnT showed an increasing trend with decreasing eGFR and with increasing severity of coronary lesion respectively (Table 2), indicating that hs-TnT may be affected by the comprehensive function of multiple factors.

In patients with CKD+STEMI, significant correlation between hs-TnT and SYNTAX Score were found only in CKD stage 2, stage 4 and in the total CKD+STEMI population, while in CKD+NSTEMI patients, there seemed no correlation between the two variables (Table 3). This may due to the limited number of patients that had coronary angiograms. Further study is strongly needed to include a larger sample size of CKD patients with coronary angiogram to verify and potentially validate this trend.

In multiple linear regression models, BNP was an independent factor affecting hs-TnT apart from ST segment elevation. Though elevated BNP in CKD was proven predominantly because of impaired renal clearance^18^, studies have reported that BNP was associated with cardiovascular disease despite of eGFR^19^,^20^,^21^. Chronic heart failure and left ventricular hypertrophy (LVH), which were common in CKD patients, may also contribute to BNP elevation^22^. Scheven’s study also indicated that BNP was not only linked to heart failure and LVH, but that there was a possible role for BNP in the diagnosis and management of myocardial ischemia^19^.

To our knowledge, our study is the first analysis that applied results of coronary angiography to examine the association between factors including AMI type and STNTAX Score with hs-TnT due to different eGFR categories, and explore optimal cutoff-values to provide better diagnostic accuracy of AMI in different stages of CKD patients. The cutoff-values may fill the gap in diagnostic value of hs-TnT in AMI patients with CKD and may facilitate early diagnosis and intervention of AMI further, improving prognosis in CKD patients.

However, the following limitations of the present study merit consideration. First, this study was conducted based on medical records from a single center with limited subjects. The diagnostic accuracy of the cutoff value needs to be validated in larger studies. Second, only hs-TnT assay was applied in our study, we could not comment on the clinical utility of other cTn assays in CKD individuals. Third, results of echocardiogram were not included in our analysis. Thus in our ongoing prospective study, we have included echocardiogram especially important factors such as left ventricular ejection fraction to clarify their relationship with hs-TnT concentration. Fourth, different timing of blood collection after symptom of chest pain may be an important factor relative to hs-TnT concentration. Li’s study reported that the cutoff-value of hs-TnT for AMI diagnosis gradually increased with the time from onset of symptoms to presentation in normal renal function patients^23^. This tendency still needs to be confirmed in CKD patients. However, due to limited sample size, we didn’t analyze time-determined hs-TnT cutoff-value under each eGFR category. Last, our study focused on relatively older patients. Thus, whether the result could be generalized in a younger population needs to be verified.

In conclusion, hs-TnT levels were associated with the type and severity of AMI, BNP and eGFR. The optimal cutoff-value of hs-TnT for diagnosis of AMI in CKD patients may be 129.45 ng/l. In different stages of CKD, eGFR-range-specific optimal cutoff-values should be considered to improve the diagnostic accuracy for AMI.

## Additional Information

**How to cite this article**: Yang, H. *et al*. Improving the diagnostic accuracy of acute myocardial infarction with the use of high-sensitive cardiac troponin T in different chronic kidney disease stages. *Sci. Rep.* **7**, 41350; doi: 10.1038/srep41350 (2017).

**Publisher's note:** Springer Nature remains neutral with regard to jurisdictional claims in published maps and institutional affiliations.

## Figures and Tables

**Table 1:** Baseline characteristics of the study population.

	Total (n = 489)	CKD+AMI group (n = 302)	CKD group (n = 187)	P
Age(y)	70.68 ± 13.43	72.70 ± 9.85	69.74 ± 10.56	0.006
Male(n/%)	280 (57.26)	183 (60.65)	97 (51.92)	NS
BMI(kg/m^2^)	21.67 ± 2.96	22.44 ± 2.99	20.2 ± 2.93	NS
Smoking(n/%)	184 (37.63)	126 (41.72)	58 (31.02)	0.021
DM(n/%)	192 (39.26)	124 (41.06)	68 (36.36)	NS
Hypertension(n/%)	384 (78.53)	241 (79.80)	143 (76.47)	NS
Dialysis(n/%)	96 (19.63)	24 (7.95)	72 (38.50)	<0.001
Hospital stay(d)	11.00 (6.00–18.00)	10.00 (5.00–16.00)	12.00 (9.00–23.00)	NS
Hb(g/l)	105.50 ± 27.86	113.96 ± 25.58	91.72 ± 25.81	<0.001
WBC(x10^9^/l)	8.71 (5.97–13.01)	8.80 (5.26–13.68)	8.36 (5.20–13.26)	NS
PLT(x10^9^/l)	164.11 ± 75.70	165.36 ± 73.73	163.00 ± 77.50	NS
Hs-cTnT(ng/l)	162.50 (47.90–1155.00)	668.20 (129.40–2716.25)	52.60 (28.45–97.75)	<0.001
BNP(pg/ml)	7081.00 (2596.00–23435.00)	7450.00 (2827.00–24428.00)	6546 (2253.75–21166.00)	NS
BUN(mmol/l)	14.06 (10.32–19.20)	13.42 (9.32–18.21)	14.84 (12.39–22.20)	NS
Cystatin-C(mg/l)	2.65 (1.97–3.81)	2.48 (1.83–3.08)	3.48 (2.35–5.21)	NS
Cr(mg/dl)	2.50 (1.85–3.99)	2.30 (1.70–3.15)	3.36 (2.08–6.21)	NS
eGFR(ml/min/1.73 m^2^)	23.88 ± 14.21	27.31 ± 14.38	18.13 ± 11.92	<0.001
30–59	37.88 ± 6.18	38.51 ± 6.47	36.29 ± 5.09	NS
15–29	22.21 ± 4.21	22.58 ± 4.12	21.32 ± 4.33	NS
<15	9.07 ± 3.41	10.87 ± 2.69	7.86 ± 3.32	<0.001
Urine protein(g/l)	0.50 (0.10–1.00)	0.30 (0.00–1.00)	0.70 (0.20–2.50)	NS
Urine WBC(/HP)	2.00 (1.00–5.00)	2.00 (1.00–5.00)	2.00 (1.00–7.00)	NS
Urine RBC(/HP)	2.00 (1.00–6.00)	2.00 (1.00–5.00)	2.00 (1.00–9.00)	NS

Abbreviations: BMI, body mass index; DM, diabetes mellitus; Hb, Hemoglobin; WBC, total leucocyte; PLT, platelet; BNP, brain natriuretic peptide; BUN, blood urea nitrogen; Cr, creatinine; eGFR, estimated glomerular filtration rate; NS, no statistical significance.

**Table 2:** hs-TnT and SYNTAX score of different AMI types under different eGFR categories.

eGFR (ml/min/1.73 m^2^)	CKD+AMI				CKD
	CKD+STEMI		CKD+NSTEMI		
	hs-TnT/n	SS/n*	hs-TnT/n	SS/n*	hs-TnT/n
60–90	608.70 (27.60–1968.00)/8	22.50 (20.00–22.50)/3	187.10 (114.20–187.10)/3	19.50/1	—/0
30–59	677.60 (131.13–3698.00)/58	15.50 (6.25–25.13)/24	468.80 (219.20–1398.00)/41	19.00 (12.00–29.00)/23	32.70 (20.30–71.80)/39
15–29	1096.50 (135.05–3157.00)/86	15.50 (9.00–22.13)/34	297.65 (54.85–1106.25)/48	15.00 (9.00–26.00)/17	51.30 (28.40–99.70)/62
<15	3075.00 (833.10–9112.00)/29	9.50 (8.25–10.75)/4	168.20 (75.45–2097.50)/29	12.00 (4.00–18.50)/5	60.60 (34.78–97.55)/86
Total	1133.00 (147.75–4013.00)/181	15.00 (9.00–22.75)/65	283.00 (105.55–1211.00)/121	17.00 (10.50–26.00)/46	52.60 (28.45–97.75)/187

Abbreviations: SS, SYNTAX Score; *number of patients had coronary angiogram.

**Table 3:** Relationship between hs-TnT and SYNTAX Score of CKD patients with different types of AMI under different eGFR categories.

eGFR (ml/min/1.73 m^2^)	CKD+STEMI			CKD+NSTEMI		
	n*	Spearman’s rho	P value	n*	Spearman’s rho	P value
Total	65	0.263	0.035	46	−0.006	0.967
60–89	3	1.000	<0.001	1	—	—
30–59	24	0.248	0.242	23	−0.100	0.650
15–29	34	0.432	0.011	17	−0.062	0.813
<15	4	0.200	0.800	5	0.500	0.391

^*^Number of patients had coronary angiography.

**Table 4:** hs-TnT concentration of smoking and non-smoking group and STEMI and NSTEMI group in patients with CKD+AMI.

hs-TnT(ng/l)			hs-TnT(ng/l)		
smoking (n = 126)	non-smoking (n = 176)	P value	STEMI (n = 181)	NSTEMI (n = 121)	P value
1130.00 (161.15–3315.25)	454.80 (107.28–2322.50)	0.011	1133.00 (147.75–4013.00)	283.00 (105.55–1211.00)	<0.001

**Table 5:** Bivariate and multivariate relationships between variables and hs-TnT of patients with CKD+AMI.

variables	Spearman’s rho	P value	Standardized β regression coefficients	P value
smoking	—	—	0.084	0.113
ST segment elevation	—	—	0.305	<0.001
BNP (pg/ml)	0.403	<0.001	0.364	<0.001
Cr (mg/dl)	0.134	0.020	0.067	0.236

Abbreviations: BNP, brain natriuretic peptide; Cr, creatinine.

**Table 6:** Optimal cutoff-values of hs-TnT in each eGFR category.

eGFR (ml/min/1.73 m^2^)	AMI (n/%)*	Optimal cutoff-value(ng/l)	Sensitivity (%)	Specificity (%)	AUC (95% CI)
total	302 (61.76)	129.45	75.2	83.2	0.839 (0.804–0.873)
30–59	99 (71.74)	99.55	82.8	82.1	0.868 (0.803–0.932)
15–29 (non-dialysis)	127 (72.57)	129.45	73.2	85.4	0.827 (0.768–0.886)
<15 (non-dialysis)	42 (60.87)	105.50	81.0	88.9	0.892 (0.819–0.965)
dialysis	24 (25.00)	149.35	79.2	81.9	0.865 (0.769–0.960)

Abbreviations: AUC, area under the curve. *Number and percentage of patients were diagnosed of AMI in each eGFR category.
